# Optimal Detection Range of RFID Tag for RFID-based Positioning System Using the *k*-NN Algorithm

**DOI:** 10.3390/s90604543

**Published:** 2009-06-10

**Authors:** Soohee Han, Junghwan Kim, Choung-Hwan Park, Hee-Cheon Yoon, Joon Heo

**Affiliations:** 1 School of Civil and Environmental Engineering, Yonsei University, Seoul 120-749, Korea; E-Mails: scivile@yonsei.ac.kr (S.H.); roak7@hanmail.net (J.K.); c142520@yonsei.ac.kr (C.P.); 2 School of Civil Engineering, Chungnam National University, Daejeon 305-764, Korea; E-Mail: hcyoon@cnu.ac.kr (H.Y.)

**Keywords:** ubiquitous positioning system, RFID, *k*-nearest neighbor, detection range

## Abstract

Positioning technology to track a moving object is an important and essential component of ubiquitous computing environments and applications. An RFID-based positioning system using the *k*-nearest neighbor (*k*-NN) algorithm can determine the position of a moving reader from observed reference data. In this study, the optimal detection range of an RFID-based positioning system was determined on the principle that tag spacing can be derived from the detection range. It was assumed that reference tags without signal strength information are regularly distributed in 1-, 2- and 3-dimensional spaces. The optimal detection range was determined, through analytical and numerical approaches, to be 125% of the tag-spacing distance in 1-dimensional space. Through numerical approaches, the range was 134% in 2-dimensional space, 143% in 3-dimensional space.

## 1. Introduction

With the advent of the ubiquitous environment, new ubiquitous positioning systems have emerged. This is due to the fact that in wireless technologies and mobile computing environments, information from a sensor network is meaningful only when the physical location of the information source is determined [1]. Ubiquitous positioning systems should be available not only for outdoor positioning but also indoor positioning environments. The drawbacks of the global positioning system (GPS), one of the most widely used positioning systems, are mispositioning in urban areas incorporating tall buildings [2] and even worse performance in indoor environments. There have been many efforts made to enhance the system, especially for indoor environments, for example, augmentation with an inertial navigation system (INS) or [“pseudo-satellite”] system [3,4]. Radio frequency identification (RFID), one of the suitable technologies for those purposes, also has been used to augment the system [5].

RFID technology uses electromagnetic energy as a medium for sending information; its two basic elements are a reader, which is connected to a host computer, and a tag. The basic concept is that the tag contains data that can be retrieved over the air by the interrogator [6]. RFID tags can be categorized as either passive or active. A passive RFID tag can operate without a power supply by reflecting an RF signal transmitted to it from the reader. In contrast, an active RFID tag requires a power supply and transmits an RF signal. Generally, a passive tag is cheaper and structurally simpler than an active tag, but the active tag provides more detection range [7]. RFID is a powerful technology, not only for automated inspection/identification of products, but also for augmenting conventional positioning systems [8,9]. If tags are attached at several known locations, an RFID reader can communicate with them, and the position of the reader, thereby, can be determined. However, the tag-installing space, which is affected mainly by the detection range of the conducted tag, is an important issue, having a decisive impact on both positioning accuracy and economy.

In an RFID-based positioning system, positions can be determined using the *k* nearest neighbor (*k*-NN) algorithm [7,10-12]. The *k*-NN algorithm has been applied also to other positioning systems such as wireless local area networks (WLAN) based positioning systems [13,14]. In *k*-NN positioning, the location of a reader can be calculated with *k*-detected reference tags the coordinates of which are known and contributed to the final value in the signal strength domain.

The objective of this study was to calculate the optimal detection range of an RFID-based positioning system in which the *k*-NN algorithm is used to determine the position of a moving reader. In other words, the optimal interval of RFID distribution for *k*-NN-based positioning was derived. However, for economical reasons, not all of the RFID tags produced are designed to provide signal strength information. Instead, they simply indicate whether a tag is detected or not within the given detection range. Moreover, the inconsistency of signal strength reception, caused by multipath and interference in the presence of obstacles, is a practical problem in *k*-NN-based positioning [7,15]. Thus, in the present study, it was assumed that no signal strength information is given. Also, reference tags were assumed to be regularly distributed in 1-dimensional, 2-dimensional and 3-dimensional spaces corresponding to various environments in the real world.

## 2. Background

### 2.1. RFID-based Positioning Using k-NN Algorithm

The *k*-NN algorithm determines the attribute of a query point by taking the weighted average of the *k* nearest neighbors to the point, and as such is a highly effective inductive inference method [16]. In RFID-based positioning using the *k*-NN algorithm, the coordinates of a target point are determined as in Equation 1:

(1) $$(x,y)=\sum_{i=1}^{k}w_{i}(x_{i},y_{i})/\sum_{i=1}^{k}w_{i}$$

In Equation 1, (*x, y*) and (*x_i_, y_i_*) are the coordinates of a target point, and the *i*-th reference point, *w_i_*, is a weight factor. The weight factor is inversely-proportional to the Euclidian distance between the reference point and the target point in the signal domain, that is, the signal strength difference between the two points. In the present study, the weight factor was simply set to *1* for detected tags and *0* for undetected ones, because the RFID system was assumed not to be provided with signal strength information for detected tags.

### 2.2. Root Mean Square Error (RMSE)

In statistics, the root mean squared error or RMSE is one of the ways to quantify the amount by which an estimator or a model differs from the true value of the quantity being estimated. The RMSE for 1-dimensional, 2- dimensional and 3-dimensional spaces can be obtained as in Equations 2 to 4:

(2) $${\text{RMSE}}_{1D}=\sqrt{\frac{\sum_{i=1}^{n}\left[{\left({\hat{x}}_{i}-x_{i}\right)}^{2}\right]}{n}}$$

(3) $${\text{RMSE}}_{2D}=\sqrt{\frac{\sum_{i=1}^{n}\left[{\left({\hat{x}}_{i}-x_{i}\right)}^{2}+{\left({\hat{y}}_{i}-y_{i}\right)}^{2}\right]}{n}}$$

(4) $${\text{RMSE}}_{3D}=\sqrt{\frac{\sum_{i=1}^{n}\left[{\left({\hat{x}}_{i}-x_{i}\right)}^{2}+{\left({\hat{y}}_{i}-y_{i}\right)}^{2}+{\left({\hat{z}}_{i}-z_{i}\right)}^{2}\right]}{n}}$$

where (*x̂_i_*), (*x̂_i_, ŷ_i_*) and (*x̂_i_, ŷ_i_, ẑ_i_*) are the estimated coordinates, (*x_i_*), (*x_i_, y_i_*) and (*x_i_, y_i_, z_i_*) are the true coordinates, and *n* is the total number of observations.

## 3. Determination of Optimal Detection Range

### 3.1. Overview of Present Study

**Figure 1.**
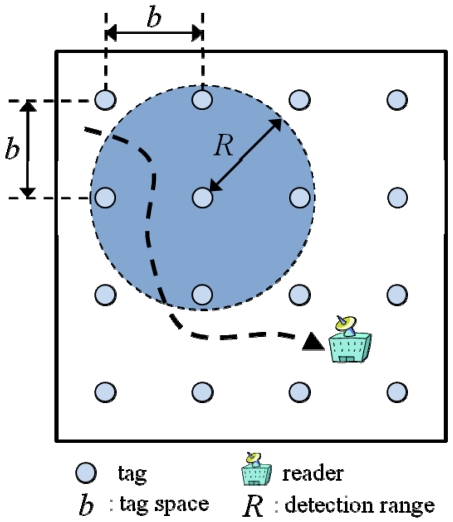
Concept of simulation in a 2-dimensional space.

For a simulated RFID-based positioning system, it was assumed that the reference tags were regularly distributed on a line in 1-dimensional space, on a regular grid in 2-dimensional space, and on a cubic lattice in 3-dimensional space. Each dimensional space could be a representative real-world space, that is, 1-dimensional space for linear objects such as industrial conveyor belts, 2-dimensional space for ordinary indoor conditions such as robot tracking, and 3-dimensional space for large-scale warehouses or construction sites. Tags within the detection range were assumed to be always detected by a reader without signal strength information. A reader was considered to move randomly in each space, and the position of the reader was determined by the *k*-NN algorithm with the same weight for each detected tag. The optimal detection range was calculated, in the analytical and numerical approaches, by minimizing the RMSE. Here, the analytical approach indicates a mathematical proof, and the numerical approach, a simulation. In 1-dimensional space, both the analytical and numerical approaches were employed. In the 2-dimensional and 3-dimensional spaces, only the numerical approach was used, owing to its complexity. Figure 1 represents the concept of the simulation in 2-dimensional space.

### 3.2. 1-Dimensional Space

#### 3.2.1. Analytical Approach

**Figure 2a.**
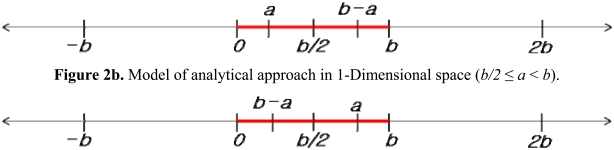
Model of analytical approach in 1-Dimensional space (*0* ≤ *a* < *b/2*). **Figure 2b**. Model of analytical approach in 1-Dimensional space (*b/2* ≤ *a* < *b*).

Figures 2a and 2b illustrate the analytical approach in 1-dimensional space. The RFID tags are evenly spaced by a tag space *b* on a line, and a reader can be regarded as moving from *0* and *b*.

Here, section [*0, b*] can stand for all of the other sections [k*b, k*(*b*+1)], where *k* is an integer, without losing generality. The detection range was defined as Equation 5, based on the assumption that the detection range should be equal to or longer than *b*:

(5) Detection range(R)=nb+a(0≤a<b)

where, *n* is a positive integer. Section [*0, b*] was divided into sub-sections and the coordinate of the detected tags can be estimated using the *k*-NN algorithm. Tables 1a and 1b show the estimated coordinate for each section.

The optimal detection range can be resolved, as shown in Equation 6, when the error term of the RMSE is minimized:

(6) $$\int_{0}^{b}{\left(x_{\text{true}}-x_{\text{measurement}}\right)}^{2}\text{dx}\to \min$$

Using the values shown in Tables 1a and 1b, Equation 6 can be rewritten as:

(7a) $$\begin{array}{l} \int_{0}^{b}{\left(x_{\text{true}}-x_{\text{measurement}}\right)}^{2}dx=\int_{0}^{a}{\left(x-0\right)}^{2}dx+\int_{a}^{b-a}{\left(x-\frac{b}{2}\right)}^{2}dx+\int_{b-a}^{b}{\left(x-b\right)}^{2}dx\\ ={\left[\frac{x^{3}}{3}\right]}_{0}^{a}+{\left[\frac{{\left(x-\frac{b}{2}\right)}^{3}}{3}\right]}_{a}^{b-a}+{\left[\frac{{\left(x-b\right)}^{3}}{3}\right]}_{b-a}^{b}=b\left[{\left(a-\frac{b}{4}\right)}^{2}+\frac{b^{2}}{48}\right] \end{array}$$

(7b) $$\begin{array}{l} \int_{0}^{b}{\left(x_{\text{true}}-x_{\text{measurement}}\right)}^{2}dx=\int_{0}^{b-a}{\left(x-0\right)}^{2}dx+\int_{b-a}^{a}{\left(x-\frac{b}{2}\right)}^{2}dx+\int_{a}^{b}{\left(x-b\right)}^{2}dx\\ ={\left[\frac{x^{3}}{3}\right]}_{0}^{b-a}+{\left[\frac{{\left(x-\frac{b}{2}\right)}^{3}}{3}\right]}_{b-a}^{a}+{\left[\frac{{\left(x-b\right)}^{3}}{3}\right]}_{a}^{b}=b\left[{\left(a-\frac{3b}{4}\right)}^{2}+\frac{b^{2}}{48}\right] \end{array}$$

**Figure 3.**
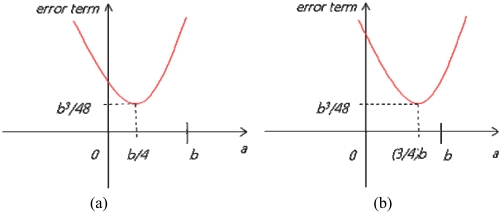
(a) Error equation graph (*0* ≤ *a* < *b/2*); (b) Error equation graph (*b/2* ≤ *a* < *b*).

Equation 7a is minimized to *b^3^/48*, as shown in Figure 3a, when *a* is *b/4* and the optimal detection range (*R*) is (*n*+*0.25*)*b*, that is, 125% of the tag-spacing distance *b* when *n* is *1*. Equation 7b is also minimized to the same value, as shown in Figure 3b, when *a* is (*3/4*)*b* and the optimal detection range (*R*) is (*n*+*0.75*)*b*, that is, 175% of the tag-spacing distance *b* when *n* is *1*.

#### 3.2.2. Numerical Approach

To verify the result of the analytical approach, the numerical approach was employed. Whereas the true positions in the analytical approach had been considered to be continuous values between *0* and *b*, the true positions in the numerical approach were considered to be the regularly sampled points between *0* and *b*, at intervals of 0.1%, in the section. For example, if tag-spacing distance *b* is 10, then 1001 points between *0* and *b* are set up as the true positions.

**Figure 4.**
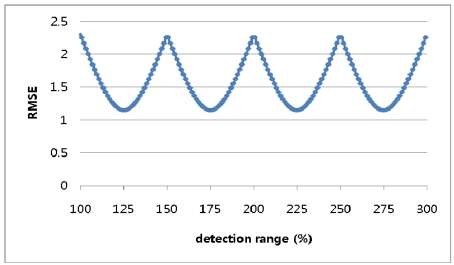
RMSE for detection ranges varying to 300% in 1-dimensional-space numerical approach.

A simulation was conducted to determine the optimal detection range with the tag space *b* set to 8 and the detection ranges varying from 100% of *b* to 300% of *b* in increments of 0.1% of *b*. Figure 4 shows a repeated curve of RMSE variation for every 50% increment in the detection range.

**Figure 5.**
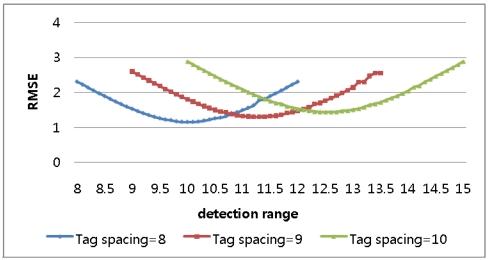
RMSE for detection ranges varying to 150% in 1-dimensional-space numerical approach.

In the result, longer detection ranges, those over 150% of *b*, were proved not to produce enhanced results. A second simulation was conducted for three different tag spaces and detection ranges varying to 150% of the tag spaces. In the result, it was shown that, as in Figure 5 and Table 2, the minimum RMSE is found when the detection range is 125% of the tag space, consistent with the analytical approach.

**Figure 6.**
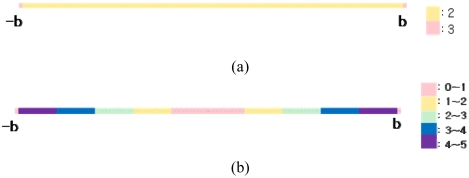
(a) Number of detected tags in worst case (b) Scale of error in worst case.

**Figure 7.**
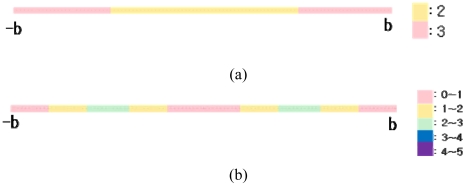
(a) Number of detected tags in best case (b) Scale of error in worst case.

The RMSE was the highest when the detection range was the same as the tag-spacing distance. Figure 6(a) shows the number of detected tags, and Figure 6(b), the scale of error at each true position in the worst case. Figure 7(a) shows the number of detected tags, and Figure 7(b), the scale of error at each true value in the best case when the detection range was optimal.

### 3.3. 2-Dimensional Space

**Figure 8.**
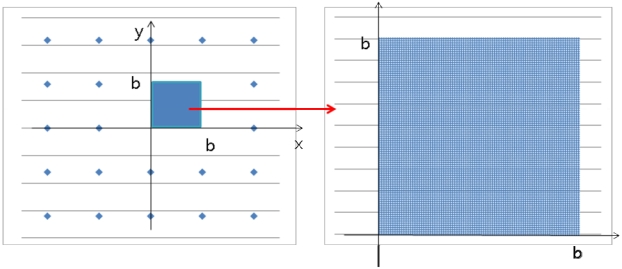
True values of simulation in 2-Dimensional space.

The true positions were considered to be the regularly sampled points between *0* and *b* at intervals of 0.1% in the section, on both the x-axis and the y-axis. Figure 8 shows the true positions for the simulations. For example, if *b* is 10, 1,001 by 1,001 points on the x-y plain are set up as the true values. The simulations were conducted with detection ranges varying from 100% of *b* to 300% of *b* along the x- and y-axes in increments of 0.1% of *b*.

**Figure 9.**
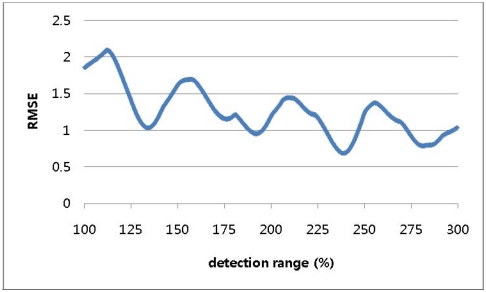
RMSE for detection ranges varying to 300% in 2-dimensional-space numerical approach.

**Figure 10.**
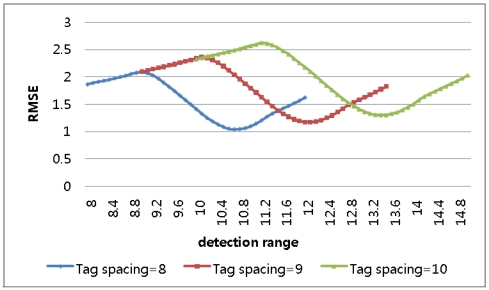
RMSE for detection ranges varying to 150% in 2-dimensional-space numerical approach.

**Figure 11.**
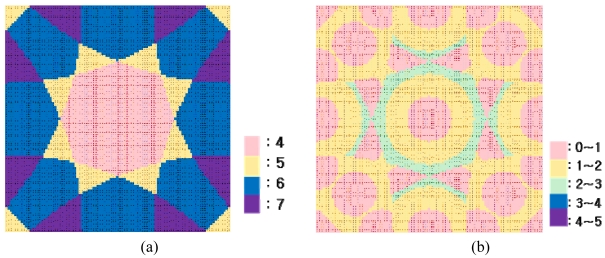
(a) Number of detected tags in worst case (b) Scale of error in worst case.

**Figure 12.**
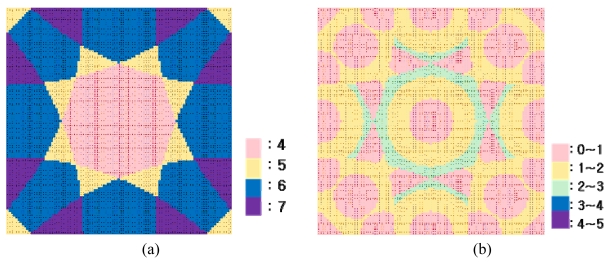
(a) Number of detected tags in best case (b) Scale of error in best case.

Figure 9 shows an RMSE fluctuation with an irregular shape, contrary to the case of the 1-dimensional space. In the result, longer detection ranges, those over 150% of *b*, were proved to be capable of producing enhanced results. However, a system with a longer detection range might cost more and it was found that there exist locally optimal detection ranges along with the local minimum RMSE. As an example of the determination of a local optimum, another simulation, more for practical economical considerations, was conducted for detection ranges varying only up to 150% of the tag spaces and with three different tag spaces. In the result, it was shown that, as shown in Figure 10 and Table 3, the minimum RMSE is found when the detection range is 134% of the tag space. Figure 11(a) shows the number of detected tags, and Figure 11(b), the scale of error at each true position in the worst case. Figure 12(a) shows the number of detected tags, and Figure 12(b), the scale of error at each true position in the best case, that is, when the detection range was optimal.

### 3.4. 3-Dimensional Space

**Figure 13.**
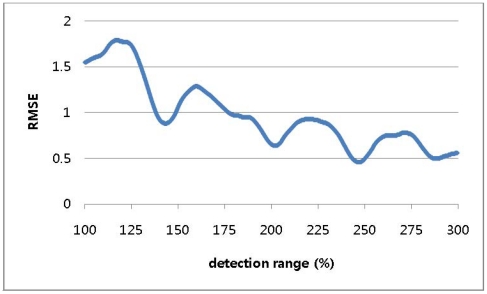
RMSE for detection ranges varying to 300% in 3-dimensional-space numerical approach.

**Figure 14.**
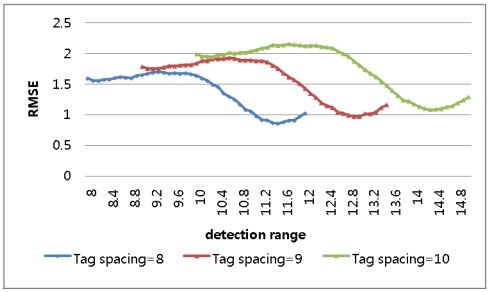
RMSE for detection ranges varying to 150% in 3-dimensional-space numerical approach.

The true positions were considered to be regularly sampled points between *0* and *b* at intervals of 0.1% on the x-, y- and z-axes. When *b* is set to 10, 1,001 by 1,001 by 1,001 points in the x-y-z cube are set up as the true positions. The simulations were conducted similarly to those for the case of the 2-dimensional space, with the z-axis added. Figure 13 shows the RMSE fluctuation to be similar to the case for the 2-dimensional space, and so an analogous analysis is possible. As in the 2-dimensional simulation, another simulation was conducted for the detection ranges varying to 150% of the tag spaces and with three different tag spaces. The result shows that, as in Figure 14 and Table 4, the minimum RMSE is found when the detection range is 143% of the tag space.

## 4. Validation of Optimal Detection Range of RFID-based Positioning System

Table 5 shows the optimal detection ranges of the RFID-based positioning system. The range was 125% of the tag-spacing distance in 1-dimensional space, 134% in 2-dimensional space, and 143% in 3-dimensional space.

**Figure 15.**
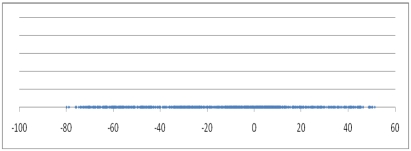
Random route with 1,000 points in 1-dimensional space.

**Figure 16.**
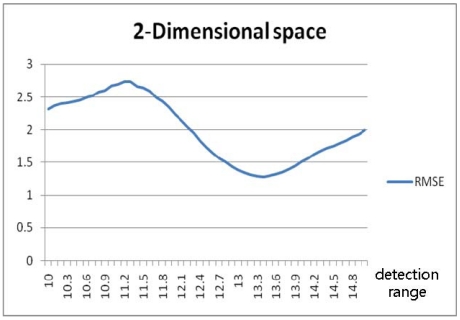
RMSE according to detection range in 1-dimensional space.

To verify these results, random routes with 1,000 points were generated in the 1-dimensional, 2-dimensional and 3-dimensional spaces and tag spacing was set to 10 in each case. Figure 15 shows the random route in the 1-dimesional space, and Figure 16 shows the optimal detection range for the random route in that space, which range was 125% of the tag-spacing distance.

**Figure 17.**
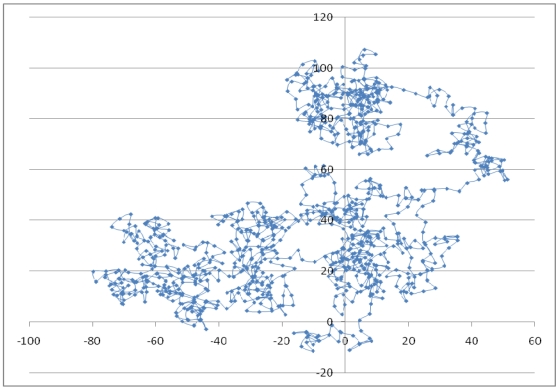
Random route with 1,000 points in 2-dimensional space.

**Figure 18.**
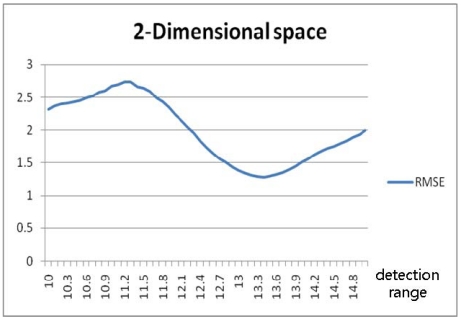
RMSE according to detection range in 2-dimensional space.

**Figure 19.**
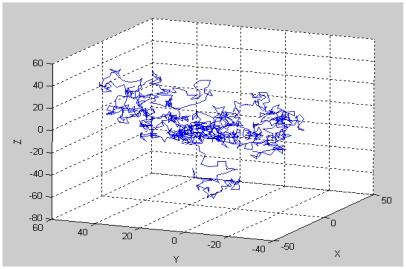
Random route with 1,000 points in 3-dimensional space.

**Figure 20.**
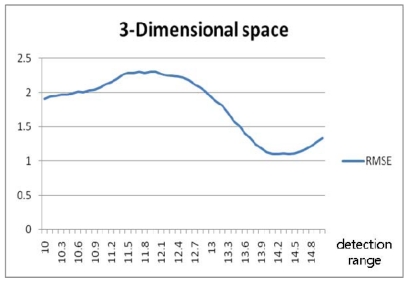
RMSE according to detection range in 3-dimensional space.

Figures 17 and 19 show the random routes in the 2- and 3-dimensional spaces, and Figures 18 and 20 show the optimal detection ranges, which were 134% of the tag-spacing distance in the 2-dimensional space and 143% in the 3-dimensional space, respectively. The results were consistent with those in the analytical and numerical approaches. So, the optimal detection range for each dimension was validated.

## 5. Conclusions and Future Study

With the advent of ubiquitous computing environments, positioning technology has become more and more important and essential, the RFID-based positioning system using the *k*-NN algorithm being one of the popular technologies. For the purposes of economy, the system was devised without provision for signal strength information. In the present study, the optimal detection ranges were determined for 1-dimensional, 2-dimensional and 3-dimensional spaces. The optimal detection range is 125% of the tag-spacing distance in 1-dimensional space, 134% in 2-dimensional space and 143% in 3-dimensional space. These results can be used as the basis for the design of RFID-based positioning systems and other applications using the *k*-NN algorithm. For example, supposing that the tag detection range is 15 m, the optimal tag interval would be 12 m in 1-dimensional space, 11 m in 2-dimensional space and 10 m in 3-dimensional space. However, in real applications, not even the distribution of tags will always be guaranteed under some conditions, as obstacles sometimes occupy the locations at which tags should be installed. Even for such cases, the present study offers a methodology for evaluating the performance of a positioning system by providing simulated RMSE between the true and estimated positions from the system.

There are additional factors to be considered, such as the detection rate for tags, the varying detection ranges of an RFID reader and signal strength information, before an accurate RFID-based positioning system design can be achieved. In future studies, tags will be installed on the basis of the findings in the present study, and will be minutely tuned and integrated with tracking simulations.

## Figures and Tables

**Table 1a.** Estimated coordinates from analytical approach (*0* ≤ *a* < *b/2*).

**Sub-section**	**detected tags**	**estimated coordinate**

*0* ≤ *x* ≤ *a*	*-nb*,…,*-b, 0, b*,…,*nb*	*0*
*a* < *x* < *b-a*	*-(n-1)b*,…,*0, b*,…, *nb*	*b/2*
*b-a* ≤ *x* < *b*	*-(n-1)b*,…,*0, b, 2b*,…,*(n*+*1)b*	*b*

**Table 1b.** Estimated coordinates from analytical approach (*b/2* ≤ *a* < *b*).


**Sub-section**	**detected tags**	**estimated coordinate**

*0* ≤ *x* < *b-a*	*-nb*,…,*-b, 0, b*,…, *nb*	*0*
*b-a* ≤ *x* ≤ *a*	*-nb*,…,*-b, 0, b, 2b*,…,*(n*+*1)b*	*b/2*
*a* < *x* < *b*	*-(n-1)b*,…,*0, b, 2b*,…,*(n*+*1)b*	*b*

**Table 2.** Optimal detection range in 1-dimensional space.

**Tag-spacing distance**	**Optimal detection range**	**Percentage**

8	10	125%
9	11.25	125%
10	12.5	125%

**Table 3.** Optimal detection range in 2-dimensional space.

**Tag-spacing distance**	**Optimal detection range**	**Percentage**

8	10.7	134%
9	12.1	134%
10	13.4	134%

**Table 4.** Optimal detection ranges in a 3-dimensional space.

**Tag-spacing distance**	**Optimal detection ranges**	**Percentage**

8	11.43	143%
9	12.9	143%
10	14.3	143%

**Table 5.** Simulation results for 1-, 2- and 3-dimensional spaces.

Dimension	Optimal detection range for tag-spacing distance
1	125%
2	134%
3	143%
